# Serum circulating sirtuin 6 as a novel predictor of mortality after acute ischemic stroke

**DOI:** 10.1038/s41598-022-23211-y

**Published:** 2022-11-28

**Authors:** Luca Liberale, Stefano Ministrini, Markus Arnold, Yustina M. Puspitasari, Thomas Pokorny, Georgia Beer, Natalie Scherrer, Juliane Schweizer, Mirjam Christ-Crain, Fabrizio Montecucco, Giovanni G. Camici, Mira Katan Kahles

**Affiliations:** 1 First Clinic of Internal Medicine, Department of Internal Medicine, University of Genoa, Genoa, Italy; 2 IRCCS Ospedale Policlinico San Martino Genoa–Italian Cardiovascular Network, Genoa, Italy; 3 Center for Molecular Cardiology, University of Zurich, Schlieren, Switzerland; 4 Internal Medicine, Angiology and Atherosclerosis, Department of Medicine and Surgery, University of Perugia, Perugia, Italy; 5 Department of Neurology, University Hospital and University of Zurich, Zurich, Switzerland; 6 Department of Endocrinology, University Hospital of Basel, Basel, Switzerland; 7 University Heart Center, Department of Cardiology, University Hospital Zurich, Zurich, Switzerland; 8 Department of Research and Education, University Hospital Zurich, Zurich, Switzerland; 9 Department of Neurology University Hospital of Basel, Basel, Switzerland

**Keywords:** Stroke, Predictive markers

## Abstract

In a murine model of acute ischemic stroke, SIRT6 knockdown resulted in larger cerebral infarct size, worse neurological outcome, and higher mortality, indicating a possible neuro-protective role of SIRT6. In this study, we aimed at evaluating the prognostic value of serum SIRT6 levels in patients with acute ischemic stroke (AIS). Serum levels of SIRT6, collected within 72 h from symptom-onset, were measured in 317 consecutively enrolled AIS patients from the COSMOS cohort. The primary endpoint of this analysis was 90-day mortality. The independent prognostic value of SIRT6 was assessed with multivariate logistic and Cox proportional regression models. 35 patients (11%) deceased within 90-day follow-up. After adjustment for established risk factors (age, NIHSS, heart failure, atrial fibrillation, and C reactive protein), SIRT6 levels were negatively associated with mortality. The optimal cut-off for survival was 634 pg/mL. Patients with SIRT6 levels below this threshold had a higher risk of death in multivariable Cox regression. In this pilot study, SIRT6 levels were significantly associated with 90-day mortality after AIS; these results build on previous molecular and causal observations made in animal models. Should this association be confirmed, SIRT6 could be a potential prognostic predictor and therapeutic target in AIS.

## Introduction

Sirtuins are a family of nicotinamide adenine dinucleotide (NAD+)-dependent enzymes with multiple biochemical functions, the main being deacetylation of lysine residues of histone and non-histone proteins^1^. In mammals, the sirtuin family is composed of seven different proteins, with different subcellular localization, tissue specificity and molecular targets^2^. Sirtuin6 (SIRT6) has nuclear distribution, is ubiquitously expressed in the body, and regulates multiple senescence-associated biological processes, such as oxidative stress, inflammation, autophagy, genome stability, telomeres elongation and glucose homeostasis^3^.

Lee et al. observed that SIRT6 knockdown by RNA silencing, enhances senescence of endothelial cells (ECs) in vitro, while reducing cell proliferation and network formation on Matrigel^®^^4^. Consistently, the expression of SIRT6 was reduced alongside senescence in ECs^5^. Although decreased SIRT6 expression may not be sufficient to induce in vivo vascular senescence, the downregulation of SIRT6 may exacerbate vascular senescence, together with external and/or additional factors such as oxidative stress^5^. Beyond senescence, SIRT6 has been shown to play a role in impaired endothelium-mediated vasorelaxation^6^, endothelial proliferation^4,7^, inflammation^8^, and atherosclerotic plaque progression^9^.

The relationship between SIRT6 and the outcome of acute ischemic stroke (AIS) was recently explored in a translational study by Liberale et al., reporting that SIRT6 knockdown is associated with larger infarct size, worse neurological outcome and higher mortality in a murine model of acute brain ischemia. In detail, SIRT6 knockdown was associated with larger permeability of the blood–brain barrier (BBB) and enhanced apoptosis, both in vivo and in vitro. Finally, downregulation of SIRT6 was associated with a worsening neurological status, assessed by the NIHSS over a time lapse of 7 days, in a small cohort of patients with AIS (n = 14)^10^.

Based on this evidence, we hypothesized that circulating levels of SIRT6 could be a predictor of post-stroke mortality in humans. Thus, we conducted an explorative analysis in a subgroup of subjects from a well-characterised previously published cohort^11^.

## Methods

### Study design and procedures

The design of the study, with inclusion and exclusion criteria, as well as the collected parameters and the handling of biological samples, were described previously (trial.gov NR NCT00390962)^11^. In brief, a total of 605 consecutive patients with a suspected cerebrovascular event were initially enrolled within 72 h from symptom onset. 362 patients had a diagnosis of AIS defined according to the World Health Organization criteria. Of those, serum was available for 317 patients which were included in the analysis (Supplementary Fig. 1). Demographic variables, comorbidities and neurological deficits assessed by a stroke neurologist using the National Institute of Health Stroke Scale (NIHSS) were collected on admission. Follow up was performed via a structured telephone interview on day 90, blinded to SIRT6 levels.

The Ethics Committee of the University Hospital Basel approved the study protocol (EKBB#157/06) and informed consent was obtained from all patients. Anonymized study data is available on reasonable request. All methods were performed in accordance with the relevant guidelines and regulations.

### Primary endpoint and sample size calculation

The primary endpoint of this analysis was mortality up to 90 days after the index event.

Based on an expected effect size of 0.735^12^ and a type I error probability < 0.05, the number of events is sufficient to achieve a statistical power of 0.8. Considering this study as a model with binary outcome, our sample size satisfied the minimum size required for a mean absolute precision error (MAPE) < 0.05 and an expected uniform shrinkage factor < 10%, when assuming a proportion of overall variance explained (R^2^_cs_) = 0.1 and a maximum number of 5 potential predictors^13^.

### Serum SIRT6 measurement

Blood was collected in EDTA containing plastic tubes, centrifuged locally at 3000*g* at 4 °C for 20 min and plasma was immediately aliquoted and frozen at − 80 °C. Serum levels of SIRT6 were measured through Enzyme-Linked Immunosorbent Assay (ELISA) with double-automated colorimetric reading, according to manufacturer’s instructions (Cusabio; Houston, TX). The lowest detection threshold was 3.12 pg/mL. Intra- and inter-assay coefficients of variation were below 8% and 10%, respectively, as described by the manufacturer.

### Statistical analysis

The statistical analysis was conducted using STATA software package, version 16 (StataCorp LLC, TX). Discrete variables are summarized as counts (percentages) and continuous variables as medians (interquartile ranges [IQR]) in the baseline characteristics. For two-group comparisons, Fisher's exact test and Mann–Whitney U test were used depending on the variable type*.* Common logarithmic transformation (base 10) was performed to normalize variables with skewed distributions (e.g. SIRT6 levels). Normality of distributions was visually assessed through Q-Q plots.

To investigate the association of SIRT6 levels with the primary endpoint, univariable and multivariable logistic regression models were constructed to calculate odds ratios and 95% confidence intervals. Potential confounders were selected among well-established predictors for AIS-related mortality: age, National Institute of Health Stroke Scale (NIHSS), chronic heart failure (CHF), atrial fibrillation (AF), and C-reactive protein (CRP). Number of covariates was limited to 5 to prevent overfitting.

To assess the incremental predictive value of SIRT6, Areas under the Receiver Operator Characteristics curve (AUROC) of the model with and without SIRT6 were compared with the likelihood ratio test.

To further investigate a possible non-linear relationship between SIRT6 levels and the outcome, additional analysis was performed, including polynomial functions. Model fit was evaluated by comparison of AUROCs of models with and without the polynomial term. Marginal effect of SIRT6 levels of the resulting model were plotted for visualisation.

Further, an optimal out-of-sample cut-off for 90-day mortality was derived using Youden`s index and applied in Cox proportional hazard regression. Kaplan–Meier curves were plotted for visualisation.

## Results

### Cohort characteristics

A total of 317 patients were included in the final analysis. Of those, 90-day follow-up was completed in 316 patients (99.7%). Median age of the cohort was 75 years [62–82], 40,5% were female. Thirty-five (11.1%) patients deceased during follow up; of them 18 (51.4%) died of stroke or its direct complications, 7 (20.0%) for other cardiovascular complications and 10 (28.6%) for other non-cardiovascular causes. Median SIRT6 levels were 989 pg/mL [797–1195]. Baseline characteristics stratified by 90-day mortality are summarized in Table 1. No significant difference was detected in baseline characteristics of the patients across the SIRT6 quartiles (Supplementary Table 1).

**Table 1:** Baseline characteristics at the time of enrolment in the overall population and after stratification for 90-day mortality.

Parameter	Total (n = 316)	90-day death		p
		No (n = 281)	Yes (n = 35)	
Age (yrs)	65 [72–82]	74 [61–81]	83 [78–87]	** < 0.001**
Sex (males)	128 (40.5)	113 (40.2)	15 (42.9)	0.760
**Medical history**				
Smoker	111 (35.1)	102 (36.3)	9 (25.7)	0.220
Hypertension	239 (75.6)	211 (75.1)	28 (80.0)	0.520
Diabetes mellitus	58 (18.4)	51 (18.1)	7 (20.0)	0.790
Hypercholesterolemia	81 (25.6)	71 (25.3)	10 (28.6)	0.670
Chronic heart failure	46 (14.6)	36 (12.8)	10 (28.6)	**0.013**
Coronary artery disease	83 (26.3)	68 (24.2)	15 (42.9)	**0.018**
Atrial fibrillation	62 (19.6)	48 (17.1)	14 (40.0)	**0.001**
Peripheral artery disease	26 (8.2)	24 (8.5)	2 (5.7)	0.570
**Clinical features**				
NIHSS	5 [2–10]	4 [2–8]	15 [8–25]	** < 0.001**
**Etiology**				
Large-artery atherosclerosis	59 (18.7)	55 (19.6)	4 (11.4)	0.357
Cardio-embolic	114 (36.1)	99 (35.2)	15 (42.9)	0.456
Small-vessel occlusion	50 (15.8)	49 (17.4)	1 (2.9)	**0.025**
Other determined etiology	16 (5.1)	15 (5.3)	1 (2.9)	0.999
Undetermined etiology	77 (24.4)	63 (22.4)	14 (40.0)	**0.035**
**Biochemical features**				
Fasting blood glucose (mmol/L)	6.1 [5.5–7.5]	6.0 [5.4–7.4]	6.2 [6.0–7.6]	0.210
Creatinine (μmol/L)	75.0 [63.0–89.0]	75.0 [62.0–88.0]	80.5 [64.0–92.0]	0.330
Total cholesterol (mmol/L)	4.38 [3.67–5.13]	4.43 [3.75–5.17]	4.06 [3.32–4.79]	0.068
HDL-cholesterol (mmol/L)	1.33 [1.09–1.99]	1.33 [1.10–1.58]	1.33 [1.04–1.73]	0.960
LDL-cholesterol (mmol/L)	2.40 [1.78–3.04]	2.45 [1.81–3.09]	2.02 [1.60–2.87]	0.082
C reactive protein (mg/dL)	3.5 [3.0–9.7]	3.1 [3.0–8.6]	13.1 [3.9–31.1]	** < 0.001**
**Medications at discharge**				
Anti-platelet agents	132 (41.6)	115(40.9)	17 (48.6)	0.468
Vitamin K antagonists	30 (9.5)	23 (8.2)	7 (20.0)	**0.034**
Statins	70 (22.1)	61 (21.7)	8 (22.9)	0.831

Data are presented as median [IQR] for continuous variables, and count (%) for categorical ones. Comparisons are performed through Mann–Whitney U test and Fisher exact test. Significant p-values are highlighted in bold.

HDL: high-density lipoprotein; LDL: low-density lipoprotein, NIH: National Institute of Health Stroke Scale.

### Association of SIRT6 serum levels with 90-day mortality

**Figure 1:**
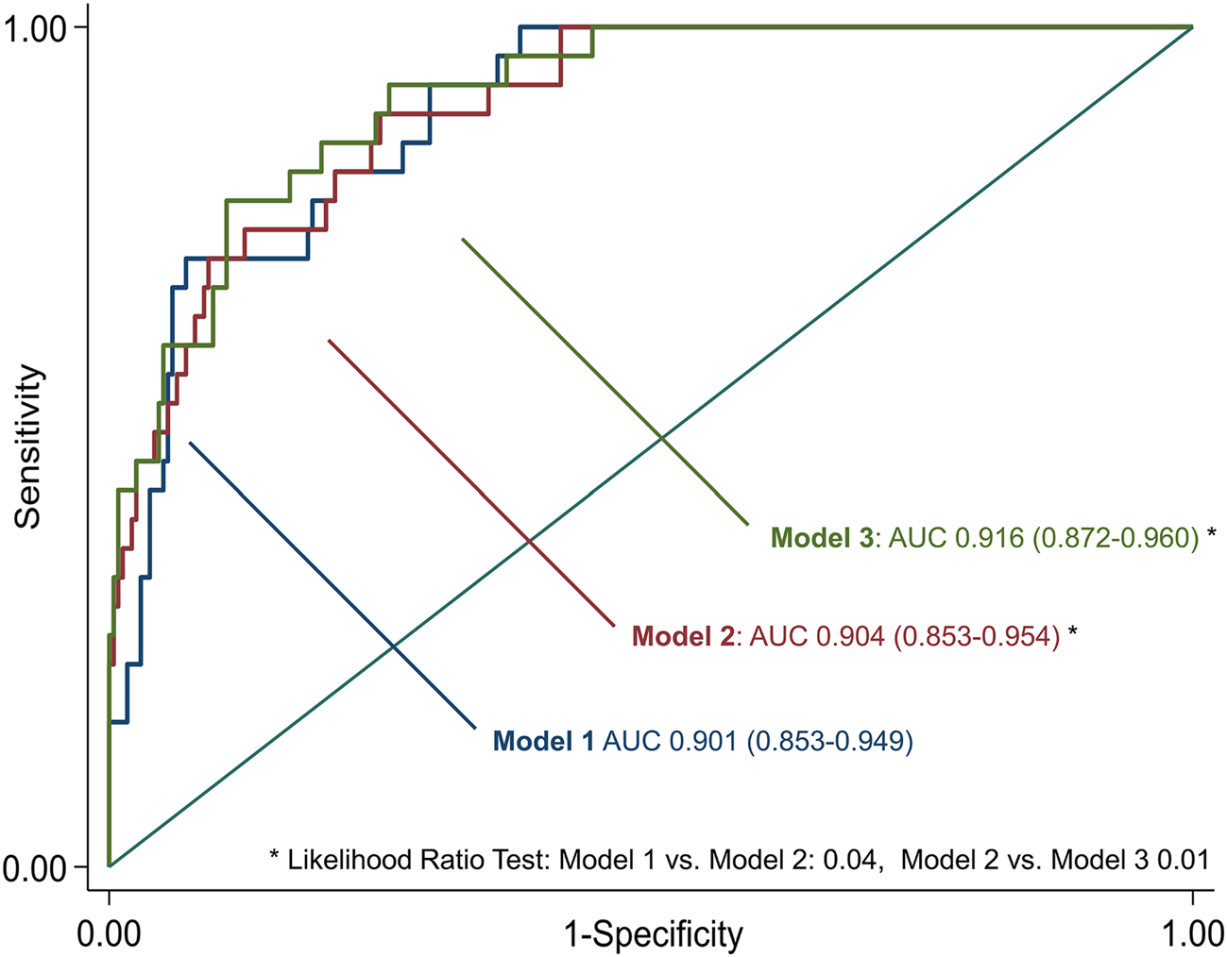
ROC curves comparing the goodness-of-fit of three different predictive models: Model 1: age, NIHSS, CHF, AF, CRP (log-transformed). Model 2: includes SIRT6 (log-transformed). Model 3: includes a second-order polynomial function of SIRT6. AF: atrial fibrillation; CHF: chronic heart failure; CRP: C reactive protein; NIHSS: National Institute of Health Stroke Scale; SIRT6: Sirtuin 6.

In univariable logistic regression, SIRT6 levels were significantly associated with 90-day mortality (OR 0.06, 95%-CI 0.01–0.52, p = 0.01, per log_10_ unit increase). This association remained significant after adjustment for well-established predictors of AIS-related mortality in a multivariable logistic regression model (OR 0.05, 95%-CI 0.01–0.99, p = 0.045, adjusted for age, sex, NIHSS, CHF, AF and CRP) (Table 2). Adding SIRT6 to the model slightly increased its predictive accuracy, with an increase in AUC from 0.901 (95%-CI 0.85–0.95) to 0.904 (95%-CI 0.85–0.96), p = 0.041 (Fig. 1). Two additional models were tested, to account for the potential prognostic role of AIS etiology and concomitant coronary artery disease (CAD).

**Table 2:** Multivariate logistic regression for 90-day mortality.

Parameter	Unadjusted			Adjusted		
	OR	95% CI	p	OR	95% CI	p
Log SIRT6 (pg/mL)	0.062	0.007–0.523	**0.011**	0.055	0.003–0.987	**0.049**
Age (years)	1.092	1.048–1.139	** < 0.001**	1.129	1.058–1.204	** < 0.001**
NIHSS	1.182	1.125–1.242	** < 0.001**	1.178	1.103–1-258	** < 0.001**
CHF	2.722	1.208–6.135	**0.016**	1.065	0.276–4.107	0.927
AF	3.236	1.538–6.811	**0.002**	0.702	0.212–2.322	0.526
Log CRP (mg/dL)	4.041	2.037–8.016	** < 0.001**	3.336	1.381–8.061	**0.007**

OR is calculated for unit increase of the independent variable (continuous variables) or for the presence of the factor (dichotomous variables). Significant p-values are highlighted in bold.

AF: atrial fibrillation; CHF: chronic heart failure; CRP: C reactive protein; NIHSS: National Institute of Health Stroke Scale; SIRT6: sirtuin 6.

As reported in Table 1, small-vessel occlusion is associated with a reduced risk of death, whereas an undetermined cause is associated with an increased risk. These differences could be mediated by the clinical severity at presentation and by systemic inflammation (Supplementary Fig. 2). So, in order to exclude co-linearity, a conservative model including only age, SIRT6, small-vessel occlusion and undetermined etiology was tested (Supplementary Table 2). However, after correction for potential confounders, only age and SIRT6 conserved their statistical significance.

Similarly, the effect of CAD was not significant after correction for the other potential confounders (Supplementary Table 3). The model including CAD has high risk of overfitting and co-linearity, since CHF is correlated to both AF and CAD. So, for the following analysis, the model was restricted to include age, sex, NIHSS, CHF, AF and CRP.

**Figure 2:**
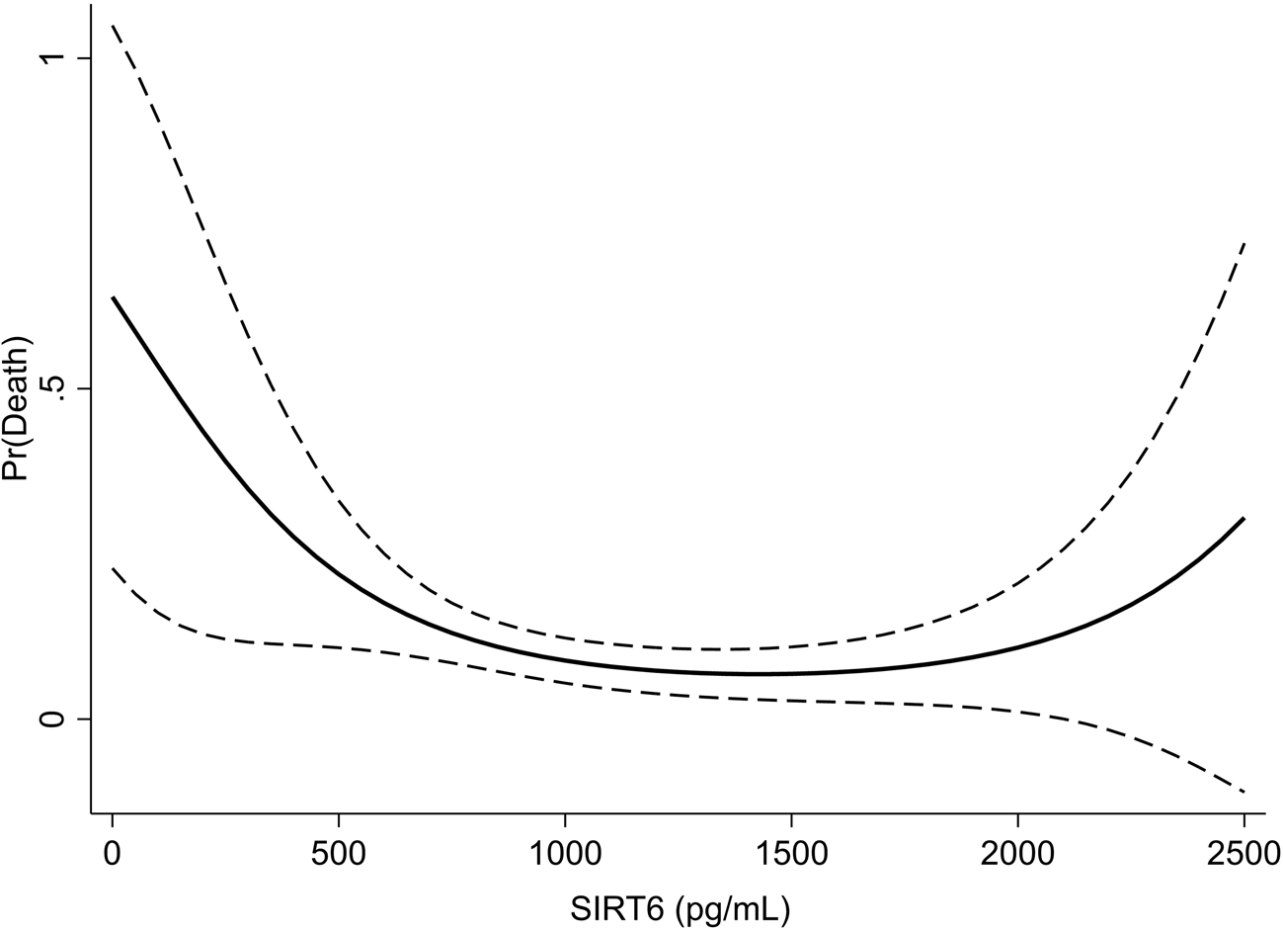
Margin effect of SIRT6 levels for the prediction of the primary outcome.

After adding the second order polynomial into the multivariable model, to account for a possible non-linear relationship between serum SIRT6 levels and 90-day mortality, the resulting model displayed a higher goodness-of-fit, compared to the model including the log-transformed SIRT-6 levels (increase in AUC from 0.904 to 0.916, p = 0.01 after correction for potential confounders; Fig. 2). The resulting marginal effects on the probability of death suggest a possible U-shaped relationship between SIRT6 levels and mortality, meaning an increased risk of death not only for low levels, but also for highest levels of SIRT6 (Fig. 2).

**Figure 3:**
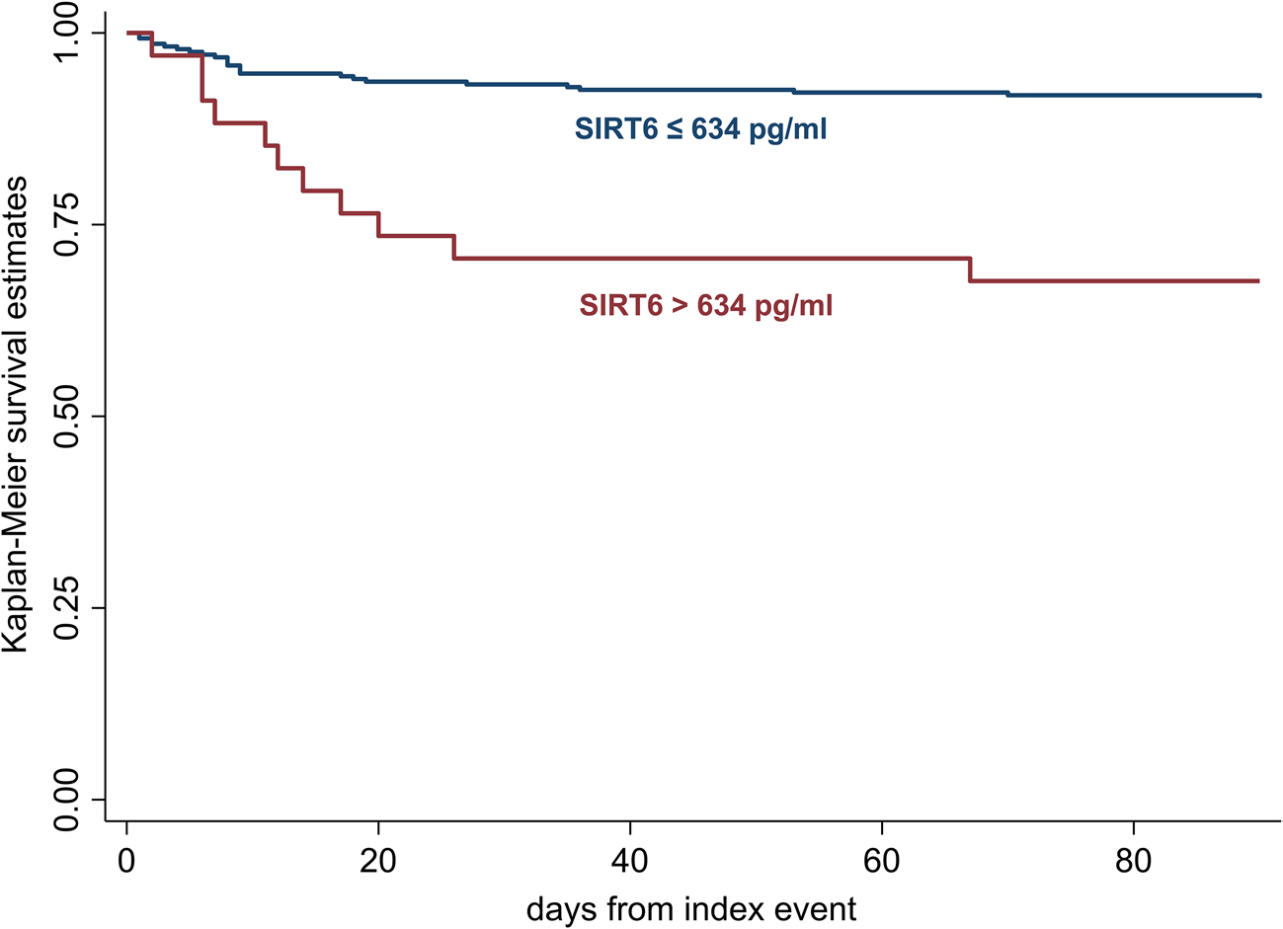
Comparison of overall survival between patients with high and low levels of SIRT6, through Kaplan–Meier curve estimates.

The optimal out-of-sample cut-off, derived by Youden’s Index, was 634 pg/mL, approximating the 10th percentile of SIRT6 levels in this cohort. Patients with SIRT6 levels below 634 pg/mL had a higher risk of death compared to patients with SIRT6 levels above 634 pg/mL (adjusted HR 5.05, 95%-CI 2.12–12.01, p < 0.001; Supplementary Table 4). Figure 3 provides Kaplan–Meier Estimates stratified by the cut-off of 634 pg/mL.

## Discussion

The results of this pilot study on serum levels of SIRT6 in patients with AIS suggest that low serum levels of SIRT6 are associated with a higher risk of death within 90 days from the event. This observation is supported by the previously reported association between low expression of SIRT6 and negative neurological outcome in a murine model of AIS^10^. The effect of SIRT6 levels on AIS-related mortality can be appreciated for very-low serum levels of SIRT6 (around the 10th percentile). As a consequence, we propose that serum levels of SIRT6 below 634 pg/mL are significantly associated with an increased risk of death, as confirmed by the Cox proportional regression analysis.

The above-described results should be considered as hypothesis-generating, rather than a conclusive evidence, because the predictive role of SIRT6 serum levels requires validation in a larger, independent cohort.

Although the role of some Sirtuins, especially SIRT1, is well-established in cardiovascular senescence and disease^14^, the evidence about the role of SIRT6 is still controversial. A low expression of SIRT6 was associated with accelerated atherosclerosis^15,16^, endothelial dysfunction^6,17^ and senescence of the endothelial cells^3–5^, and therefore proposed as a predictor of coronary artery disease^12^ and a negative prognostic factor in myocardial infarction^18,19^. However, to the best of our knowledge, this is the first report to describe the association between SIRT6 and post-stroke mortality in humans. In the above-mentioned pre-clinical study, SIRT6 knockdown was associated with increased apoptosis of endothelial cells in the brain microcirculation and, consequently, increased permeability of the blood–brain barrier^10^. This is a key process determining reperfusion damage in acute ischemic stroke^20^ and could therefore contribute to the higher mortality of patients, observed in the present study. SIRT6 is also involved in additional pathophysiological processes, that could explain the increased risk of death among patients presenting very-low serum levels. SIRT6 expression was associated to the suppression of pro-inflammatory pathways^21,22^ and, therefore, SIRT6 reduction promotes systemic inflammation^23^ and neuroinflammation after an acute brain damage^24,25^. Inflammation has a detrimental role on the natural course of AIS^26^, as confirmed by the independent association between CRP and mortality herein reported. Interestingly, the association between SIRT6 and mortality was independent of CRP, indicating that systemic inflammation is not the unique mechanism driving this association. Ultimately, SIRT6 could have a neuroprotective role in the setting of acute brain ischemia. In general, low levels of SIRT6 are associated with a higher susceptibility to neurons degeneration in different neurodegenerative diseases^27^. Besides, the antioxidant effect of SIRT6 has been found to protect neurons from the ischemia/reperfusion injury^28^. In this regard, we must acknowledge that the current study does not specifically tackle neuroprotection, since we did not show any association between SIRT6 and neurological functional outcome. In this regard, future studies focusing on ischemic volume and neurological outcomes are needed.

Additional analysis, including a second order polynomial function to account for possible non-linear association, suggests that the relationship between SIRT6 and AIS-related mortality could be indeed U-shaped, with an increasing risk of death not only for very-low, but also very-high levels of SIRT6. Comparing AUROCs of resulting models confirmed that the non-linear model relationship performed significantly, although only slightly, better than the one restricted to a linear relationship. To the best of our knowledge, no previous clinical or pre-clinical study described an association between very-high levels of SIRT6 and any cardiovascular or neurological disorder. Results of the translational study from Liberale et al.^10^ suggests that SIRT6 prevents endothelial apoptosis, thus maintaining the integrity of the blood–brain barrier and preventing the progression of the damage caused by its leakiness. However, translational studies on solid tumors observed that high levels of SIRT6 may have a pro-apoptotic effect too^29,30^. Whether these results can be replicated in vascular tissue is still unclear, therefore, further studies in pre-clinical models, as well as independent cohorts of patients, are needed to clarify the potential role of very-high SIRT6 levels in AIS.

Early anticoagulation in patients with AF and AIS is strongly recommended in current guidelines^31^, to reduce the risk of AIS recurrence. Surprisingly, we found that patients receiving anticoagulants at discharge, displayed also a higher 90-day mortality. However, this result must be cautiously pondered. First of all, present data were collected before the introduction of direct oral anticoagulant agents, so they do not portray the current state-of-art in the management of AF. Furthermore, this result is highly influenced by the concomitant presence of AF itself, which is a negative prognostic factor.

The main limitations of the present study are represented by the small sample size, and in particular the small incidence of events (11.1%), which prevented us from performing a broad and thorough analysis of potential confounders. Besides, data about the acute treatment of the stroke were not available. Furthermore, we did not include a control group of patients without AIS in the analysis, so we cannot rule out a potential overlap between the values that we associated to an increased risk of negative outcome and values in a healthy population. Finally, the current lack of an independent cohort prevented us from validating the predictive role of SIRT6 on the risk of AIS-related death.

Should the association between SIRT6 and AIS-related mortality be confirmed, circulating levels of SIRT6 could help stratifying the risk of patients with AIS, in order to optimize the management of the patients with highest risk. Furthermore, multiple potential activators of SIRT6 are currently under investigation^32^ and some of these molecules could be successfully trialled in the future, also in AIS. Indeed, no specific treatment is currently available to improve the prognosis of patients with AIS, beyond systemic thrombolysis and/or mechanical thrombectomy, and this is the most urgent clinical need in this field.

In conclusion, SIRT6 levels, determined within 72 h from the onset of symptoms, are associated with 90-day mortality after AIS. These results confirm previous observations in animal models in a well-characterized patient cohort. Further studies are needed to validate the potential value of SIRT6 as a predictive biomarker in an independent larger validation cohort.

## Supplementary Information

Supplementary Information.

## Data Availability

Original database is available on motivated request by the corresponding Author.
