# Supplementary material for: Serum circulating sirtuin 6 as a novel predictor of mortality after acute ischemic stroke
Source: Sci Rep. 2022 Nov 28;12:20513. doi: 10.1038/s41598-022-23211-y (PMC9705558; doi:10.1038/s41598-022-23211-y)
Supplement: Supplementary file 1 — Supplementary Information. [file 41598_2022_23211_MOESM1_ESM.docx]

**Supplementary Material**

**Supplementary Table 1.** Baseline characteristics at the time of enrolment in the overall population and divided according to Sirtuin 6 quartiles

| **Parameter** | **Q1**  **(n=79)** | **Q2**  **(n=80)** | **Q3**  **(n=79)** | **Q4**  **(n=79)** | **p** |
| --- | --- | --- | --- | --- | --- |
| Age (yrs) | 75 (64-83) | 73 (61-81) | 75 (67-83) | 75 (61-81) | 0.293 |
| Sex (males) | 30 (38.0%) | 27 (33.8%) | 37 (46.8%) | 34 (43.0%) | 0.355 |
| *Medical history* |  | | | | |
| Smoker | 33 (41.8%) | 28 (35.0%) | 27 (34.2%) | 24 (30.4%) | 0.506 |
| Hypertension | 59 (74.7%) | 58 (72.5%) | 64 (81.0%) | 59 (74.7%) | 0.627 |
| Diabetes mellitus | 18 (22.8%) | 17 (21.3%) | 8 (10.0%) | 15 (19.0%) | 0.166 |
| Hypercholesterolemia | 23 (29.1%) | 24 (30.0%) | 20 (25.3%) | 14 (17.7%) | 0.272 |
| Chronic heart failure | 11 (13.9%) | 7 (8.8%) | 15 (19.0%) | 13 (16.5%) | 0.298 |
| Coronary artery disease | 24 (30.4%) | 23 (28.7%) | 14 (17.7%) | 22 (27.8%) | 0.258 |
| Atrial fibrillation | 17 (21.5%) | 14 (17.5%) | 13 (16.5%) | 18 (22.8%) | 0.702 |
| Peripheral artery disease | 5 (6.3%) | 7 (8.8%) | 4 (5.1%) | 10 (12.7%) | 0.319 |
| *Clinical features* |  | | | | |
| NIHSS | 5 (3-12) | 5 (2-9) | 5 (2-10) | 5 (2-10) | 0.620 |
| *Etiology* |  |  |  |  |  |
| Large-artery atherosclerosis | **15 (19.0%)** | **16 (20.0%)** | **12 (15.2%)** | **16 (20.3%)** | **0.835** |
| Cardio-embolic | 28 (35.4%) | 26 (32.5%) | 27 (34.2%) | 33 (41.8%) | 0.639 |
| Small-vessel occlusion | 11 (13.9%) | 17 (21.3%) | 12 (15.2%) | 10 (12.7%) | 0.456 |
| Other determined etiology | 3 (3.8%) | 6 (7.5%) | 5 (6.3%) | 3 (3.8%) | 0.653 |
| Undetermined etiology | 22 (27.8%) | 15 (18.8%) | 23 (29.1%) | 17 (21.5%) | 0.361 |
| *Biochemical features* |  | | | | |
| Fasting blood glucose (mmol/L) | 6.3 (5.7-8.2) | 6.1 (5.6-7.4) | 5.8 (5.1-6.7) | 6.4 (5.3-7.8) | 0.226 |
| Creatinine (μmol/L) | 71 (60-82) | 79 (69-90) | 73 (62-91) | 76 (59-90) | 0.134 |
| Total cholesterol (mmol/L) | 4.42 (3.78-5.16) | 4.45 (3.49-5.04) | 4.49 (3.94-5.10) | 4.25 (3.65-5.46) | 0.658 |
| HDL-cholesterol (mmol/L) | 1.36 (1.08-1.68) | 1.24 (0.99-1.52) | 1.39 (1.20-1.68) | 1.31 (1.11-1.54) | 0.110 |
| LDL-cholesterol (mmol/L) | 2.44 (1.71-3.01) | 2.38 (1.84-2.94) | 2.47 (1.78-3.09) | 2.21 (1.78-3.35) | 0.711 |
| C reactive protein (mg/dL) | 4.2 (3.0-9.6) | 3.6 (3.0-10.4) | 3.0 (3.0-7.6) | 3.5 (3.0-10.8) | 0.925 |

HDL: high-density lipoproteins; LDL: low-density lipoproteins; NIHSS: National Institute of Health Stroke Scale

**Supplementary Table 2.** Multivariable logistic model for 90-day mortality, including etiology of the acute ischemic stroke.

| **Parameter** | **OR** | **95% CI** | **p** |
| --- | --- | --- | --- |
| Log SIRT6 (pg/mL) | 0.059 | 0.006-0.630 | **0.019** |
| Age (years) | 1.094 | 1.048-1.143 | **<0.001** |
| Small-vessel occlusion | 0.134 | 0.017-1.058 | 0.057 |
| Undetermined etiology | 1.368 | 0.610-3.069 | 0.447 |

OR is calculated for unit increase of the independent variable (continuous variables) or for the presence of the factor (dichotomous variables). Significant p-values are highlighted in bold.

SIRT6: sirtuin 6.

**Supplementary Table 3.** Multivariable logistic model for 90-day mortality, including the effect of coronary artery disease (CAD)

| **Parameter** | **Unadjusted** | | | **Adjusted** | | |
| --- | --- | --- | --- | --- | --- | --- |
|  | **OR** | **95% CI** | **p** | **OR** | **95% CI** | **p** |
| Log SIRT6 (pg/mL) | 0.062 | 0.007-0.523 | **0.011** | 0.058 | 0.003-0.975 | **0.048** |
| Age (years) | 1.092 | 1.048-1.139 | **<0.001** | 1.131 | 1.057-1.210 | **<0.001** |
| NIHSS | 1.182 | 1.125-1.242 | **<0.001** | 1.190 | 1.109-1.276 | **<0.001** |
| CHF | 2.722 | 1.208-6.135 | **0.016** | 0.805 | 0.202-3.209 | 0.758 |
| AF | 3.236 | 1.538-6.811 | **0.002** | 2.551 | 0.891-7.303 | 0.081 |
| CAD | 2.349 | 1.140-4.841 | **0.021** | 0.695 | 0.206-2.348 | 0.559 |
| Log CRP (mg/dL) | 4.041 | 2.037-8.016 | **<0.001** | 3.188 | 1.317-7.719 | **0.010** |

OR is calculated for unit increase of the independent variable (continuous variables) or for the presence of the factor (dichotomous variables). Significant p-values are highlighted in bold.

AF: atrial fibrillation; CHF: chronic heart failure; CRP: C reactive protein; NIHSS: National Institute of Health Stroke Scale; SIRT6: sirtuin 6.

**Supplementary Table 4.** Cox proportional regression for 90-day mortality.

| **Parameter** | **Unadjusted** | | | **Adjusted** | | |
| --- | --- | --- | --- | --- | --- | --- |
|  | **HR** | **95% CI** | **p** | **HR** | **95% CI** | **p** |
| SIRT6 ≤634 pg/mL | 4.276 | 2.093-8.737 | **<0.001** | 5.042 | 2.122-11.979 | **<0.001** |
| Age (years) | 1.083 | 1.044-1.123 | **<0.001** | 1.093 | 1.041-1.148 | **<0.001** |
| NIHSS | 1.126 | 1.095-1.157 | **<0.001** | 1.118 | 1.073-1.165 | **<0.001** |
| CHF | 2.581 | 1.240-5.375 | **0.011** | 1.144 | 0.402-3.260 | 0.801 |
| AF | 2.998 | 1.524-5.897 | **0.001** | 0.802 | 0.282-2.282 | 0.679 |
| Log CRP (mg/dL) | 3.538 | 1.989-6.293 | **<0.001** | 2.981 | 1.563-5.685 | **<0.001** |

HR is calculated for unit increase of the independent variable (continuous variables) or for the presence of the factor (dichotomous variables). Significant p-values are highlighted in bold.

AF: atrial fibrillation; CHF: chronic heart failure; CRP: C reactive protein; NIHSS: National Institute of Health Stroke Scale; SIRT6: sirtuin 6.

**Supplementary Figure 1.** Flow-chart of data inclusion in the study.


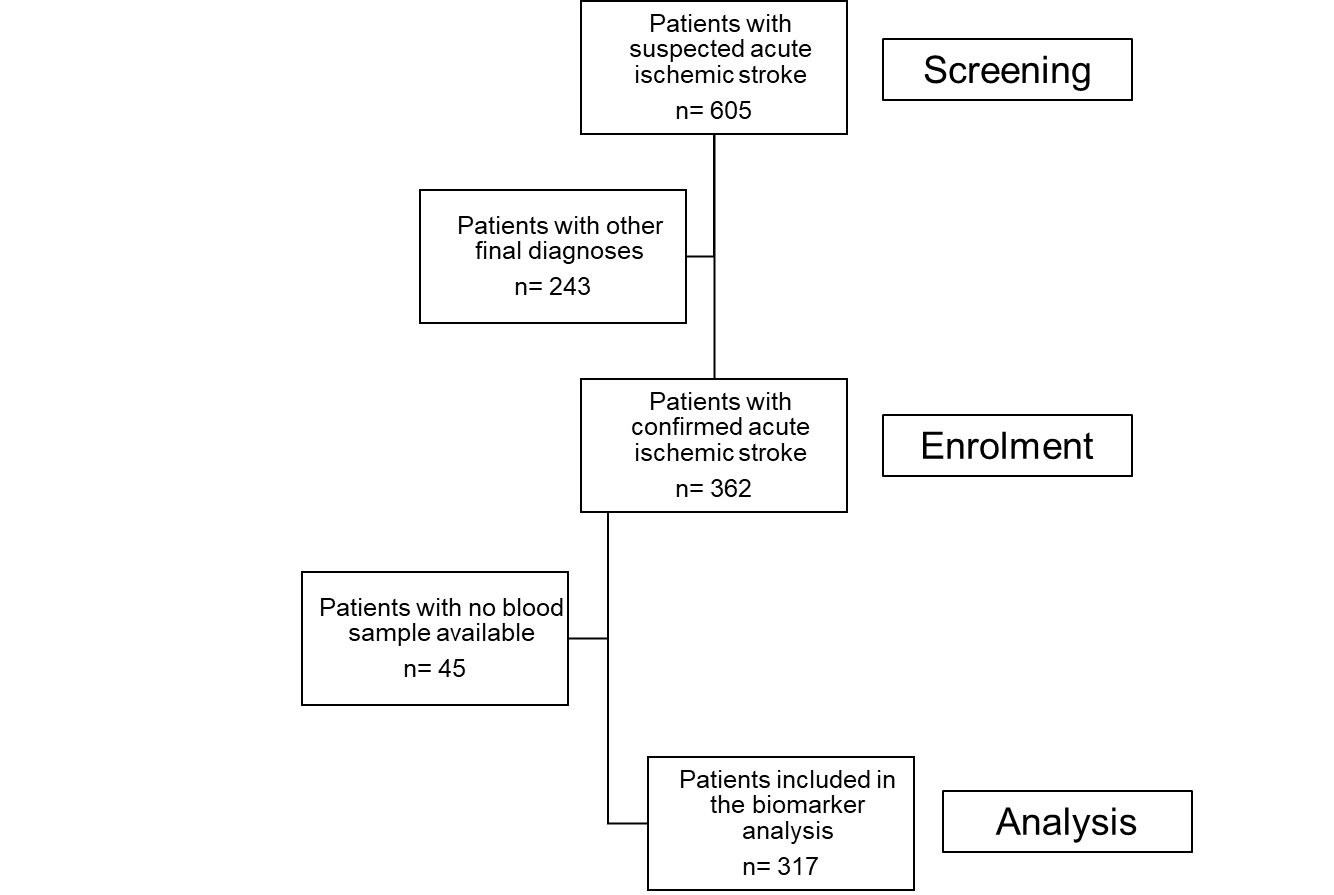


**
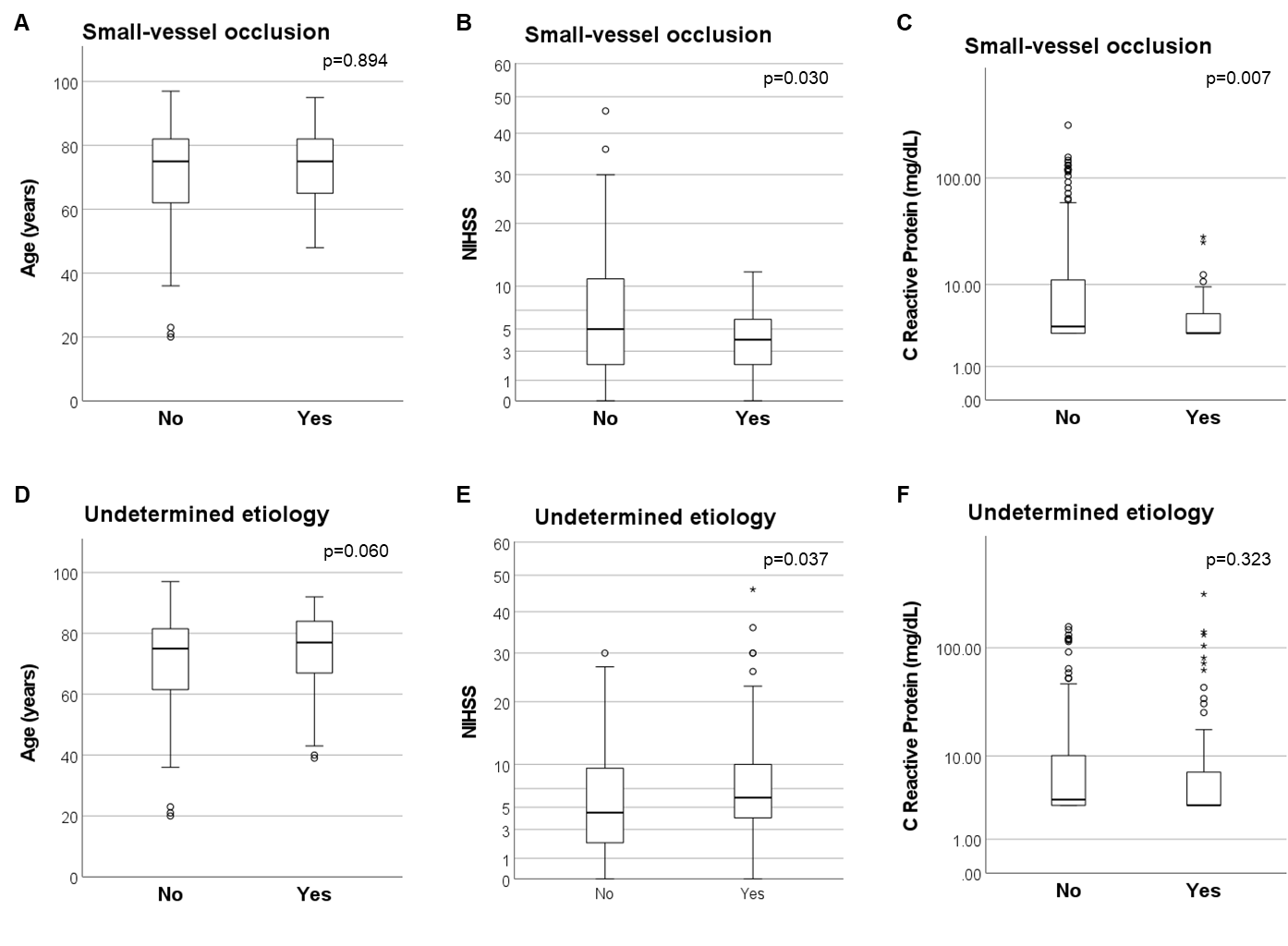
Supplementary Figure 2.** Comparison of main determinants of mortality risk (age, National Institute of Health Stroke Scale – NIHSS, and C reactive protein), according to ischemic stroke etiology. **A-C.** Differences between patients with and without small-vessel occlusion. **D-F.** Differences between patients with any determined etiology and undetermined etiology.

**Supplementary Figure 3.** Correlation matrix of chronic heart failure (CHF), atrial fibrillation (AF) and coronary artery disease (CAD). Spearman’s rank correlation was employed.

**
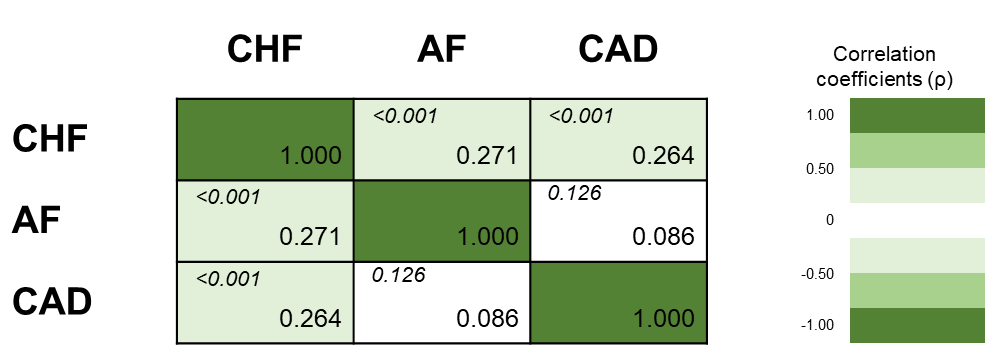
**
